# The Surgery of Nephritis1Read before the Rush County (Ind.) Medical Society, May 2, 1904.

**Published:** 1905-04

**Authors:** J. C. Sexton

**Affiliations:** Rushville, Ind.


### The Surgery of Nephritis.[1]

By J. C. SEXTON, M. D., Rushville, Ind.

[*Cincinnati Lancet-Clinic*, May 28, 1904.]

IN OPENING the discussion today upon the surgical treatment of chronic nephritis, I wish to allude briefly to three cases. These cases are not alike, but they illustrate certain points that will come up for discussion. On July 4, 1884, I saw an Irishman, 24 years old, in uremic convulsions all day. He had been pale and dropsical, unable to work on account of weakness and heart palpitation for two years. For two days prior to the convulsions he had complete suppression of urine. The spasms were very severe. He did not come to consciousness between attacks, and in the twenty-four hours he had nearly thirty seizures. We fully expected him to die, but his kidneys again resumed their function and he made a tedious recovery. That was in 1884. This man was still living in 1896. He had albumin and casts in the urine. To my positive knowledge he had chronic nephritis, with casts and large quantities of albumin in the urine, for fourteen years. He moved to another part of our state and may be still living for all I know.

I fixed a movable right kidney, making a rather free but not complete decapsulation, for a young lady, in December, 1901.

1. Read before the Rush County (Ind.) Medical Society, May 2, 1904.

She came for operation for prolapsed left kidney in December, 1902. After completing the operation on the left side I took the opportunity to dissect down upon the right kidney, and was able to demonstrate to the full satisfaction of the physicians present not only the number and size of the adhesions, but also—and this I ask you to note particularly—their extreme vascularity.

Eleven months ago I decapsulated the kidneys of a young lady for Bright's disease. This patient was sent to me by Dr. Green. The diagnosis of Bright's disease had been made by Dr. Arnold, of Colorado Springs, Dr. Green, of Rushville, and Dr. E. E. Hamilton, of Connersville. She had an enlarged right kidney, prolapsed to the second degree, and had carried undoubted symptoms of chronic nephritis for three years. She was pale and weak. Her hemoglobin, 70 per cent. She had edema of face, hands and feet. She could scarcely walk a block, and could not go up stairs at all on account of palpitation and dyspnea. She hardly knew herself without headache, and had frequent attacks of vomiting. There were days when she would eliminate scarcely any urea at all, and during these days her headaches and gastric symptoms were always worse. Then, again, there were days when she would eliminate from five to eight grains of urea per ounce of urine. The symptoms and character of the casts, as well as the condition of the organs at the time of operation, denoted a chronic parenchymatous nephritis, probably undergoing changes toward the interstitial variety. It is now a year since she came under the care of Dr. Green, and I can say that all her symptoms have improved, while most of them have entirely disappeared. This patient had been under treatment part of the time and under strict diet all the time for a year and a half preceding her operation, but was steadily growing worse.

The subject of treatment of the various forms of chronic nephritis by surgical measures has been before the profession now for some years, and a good bit of evidence both for and against the idea of operative interference has been presented.

The philosophy of the operation, as first held and still held by some, is that division and removal of the fibrous capsule relieves tension, and thus the inflammation is overcome. The opinion promulgated by Dr. Edebohls, of New York, is not that the inflammatory process is checked by relief of tension, but that a new blood supply is afforded to the crippled and failing organs through the vascularity of the adhesions that result from exposing the raw cortex of the kidneys. All forms of chronic nephritis have been thus treated, and if it becomes established that under the influence of the new blood supply the destruction can be checked, or even, as some claim, a regeneration of kidney epithelium takes place, patients with Bright's disease in all its different forms will doubtless be benefited.

It does not seem to me to make any difference what view one may hold as to the pathology, for all forms of chronic kidney inflammation have one common lesion—namely, interference with and blocking up of, or compression upon, the arterioles and a gradual cutting off of the blood supply. One man may believe that all arterio-capillary sclerosis is due to the failure of the kidneys to eliminate certain toxins that irritate the intima of the arterioles of the body. Another thinks that arterio-capillary fibrosis or sclerosis is due to other causes, and that the changes found in the kidneys are only part and parcel of a general disease. In either instance the restoration or preservation of such vital organs as the kidneys must be of undoubted benefit. So we may say that interstitial nephritis, parenchymatous nephritis, chronic diffuse nephritis with or without exudation of serum-albumin, glomerulo-nephritis—in short, all forms of kidney inflammation—tend to destroy the kidney by encroachment upon its bloodvessels, and the idea of the surgical interference is to bring to these organs a new blood supply.

Now the question arises, will the kidney get new blood currents from these adhesions? I think it will, for I have seen in the case above reported the extreme vascularity of adhesions one year old, and because I cannot explain in any other way the undoubted cures of some of these cases. Some of the kidneys decapsulated for chronic nephritis have stood the test of pregnancy, of years, and of acute infectious disease. Every one knows a case of Bright's may live long and die of something else. I am only through telling you of one fourteen years sick, and the operation must do better than merely not kill, or it is of no real advantage. I have never seen a case of interstitial nephritis or chronic parenchymatous nephritis get well. I know cases that have lived many years, and even die of some other disease, but they carried crippled kidneys all the time.

On the other hand, there has been presented contradictory evidence. It has been claimed that experiments on animals show that in eight or ten months after a decapsulation the scar tissue over the cortex forms a new fibrous capsule, dense and firm, shutting off the new blood currents springing from the adhesions; then, again there is no evidence to show that there is any deep penetration of the cortex by new vessels. Opposition to the operation has been presented because it is said to be not infrequently fatal, and the cures are thus offset by the fatalities.

The operation, I can say, is not difficult, and should not be dangerous. If it is attempted upon small, red, contracted kidneys, it may be impossible, and would do no good if it was possible, for the kidney under such pathology is practically gone. On the other hand, if a kidney can be delivered without violence to its structure its capsule can be removed and the risk be no greater than for nephrorrhaphy, which practically has no mortality.

Therefore, I submit that we have enough evidence of the benefit to be derived from decapsulation to warrant at least a consideration of this operation in every case. I think patients should be kept upon careful treatment and diet afterwards, for there would be no sense in throwing extra work upon organs just because they have been recently operated. One must not expect to see very rapid changes in the health of these patients. The albumin may not disappear for a year. Edebohls does not look for much improvement before six months, or until the new anastomosis is established. Yet it is, to say the least, sometimes surprising how rapidly changes take place in the urine and improvement begins. In the case of Bright's just reported, in which I operated on the kidneys at intervals of a month, segregation of the urines on the twenty-fourth day showed three times as much urea from the operated kidney as from the one not yet decapsulated. Both these kidneys, however, were swollen, and the early improvement may have been due in part to relief of tension. I wanted to wait longer for the second operation until repeated tests would show the certain effect, but my patient experienced such marked relief from pain that she insisted upon the second operation.

Operations for chronic nephritis have shown that the disease is not infrequently unilateral, or that only limited portions of the organs are involved.

Having expressed my belief in the utility and safety of this treatment, and that operations should be given consideration in every case, yet that is quite a different thing from saying that the operation is indicated in every case. I am satisfied that Dr. Edebohls made a mistake to operate on all the thirty-two cases that presented themselves in 1902, many of which—the majority, in fact—were complicated by vascular and cardiac lesions. His death-rate of thirteen in fifty-one cases is altogether too high, and he must have operated on some cases in no way fit and in no way to be benefited by any treatment.

"Ferguson believes that decapsulation in acute, subacute and chronic nephritis, both interstitial and parenchymatous, is a practical and effectual procedure, but is not a panacea." (Murphy, Year-Book.) He thinks the beneficial result due to relief of tension and establishment of collateral circulation with the diseased cortex.

Schmidt (*Medical Record,* 1902,) denies *in toto* that decapsulation in any way inhibits the progress of Bright's disease, but admits that surgical interference has been successful in relieving pain and hemorrhage when medication had utterly failed. In anuria consequent upon or uremic symptoms occurring in the course of Bright's disease, incision and reflection of the capsule has been attended with relief, but this author thinks the effect due to the immediate relief of tension, and in no way permanent.

At the meeting of the American Medical Association in New Orleans, Ferguson, Edebohls, Gibbons and Winslow reported cures of chronic nephritis by decapsulation.

Horwitz reported (*American Medicine,* 1903,) four cases of chronic nephritis, all of which were symptomatically cured.

Johnson (*Annals of Surgery,* 1903,) performed the operation on fifteen dogs. He killed and examined these dogs at various periods after the operation. The kidneys were found surrounded by scar tissue, and in no instance could he discover any considerable anastomosis between renal and perirenal vessels. Edebohls, on the other hand, examined a kidney which he had decapsulated four months earlier, and found greatly enlarged bloodvessels entering the kidney from the fatty capsule.

Blake (*Boston Medical and Surgical Journal,* 1902,) had five operations with two cures, two deaths and one unimproved. Caille had marked improvement in a severe case of chronic nephritis in a five-year-old child after operation by Edebohls. Cases such as those reported by Whitacre, Bevan, Mendoza and Lucas show the operation to be of undoubted value in anuria from various causes. The reviews by Murphy, Coley, and Belfield clearly show that while the theory of renal and peritoneal anastomosis is not yet established, and most of us are far from accepting the idea of regeneration of kidney epithelium, yet there have been such a number of striking improvements and such a number of cures in interstitial nephritis, as well as in other forms of Bright's disease, that we can claim an advance in kidney surgery, and can present for consideration to every physician treating a case of Bright's disease or other chronic inflammation of the kidneys an operation that has merit, that has wrought cures, that is or should be practically without mortality, and one which gives the patient an advantage not offered by any other plan of treatment.

---

A SOCIETY of women in Berlin has recently presented a petition to the Prussian Minister of Education, praying for the prohibition of corsets in young ladies' schools on the ground that this garment is prejudicial to the health of the growing girl.

